# Numerical Investigation of Multifunctional Plasmonic Micro-Fiber Based on Fano Resonances and LSPR Excited via Cylindrical Vector Beam

**DOI:** 10.3390/s21165642

**Published:** 2021-08-21

**Authors:** Min Liu, Lan Yu, Yunze Lei, Xiang Fang, Ying Ma, Lixin Liu, Juanjuan Zheng, Ke Lin, Peng Gao

**Affiliations:** 1 School of Physics and Optoelectronic Engineering, Xidian University, Xi’an 710071, China; lium@xidian.edu.cn (M.L.); 20051110305@stu.xidian.edu.cn (L.Y.); yunze.lei@stu.xidian.edu.cn (Y.L.); 20051212230@stu.xidian.edu.cn (X.F.); yingma@xidian.edu.cn (Y.M.); lxliu@xidian.edu.cn (L.L.); jjzheng@xidian.edu.cn (J.Z.); klin@xidian.edu.cn (K.L.); 2 Guangzhou Institute of Technology, Xidian University, Guangzhou 510555, China

**Keywords:** plasmonic micro-fiber, Fano resonances, localized surface plasmon resonance, cylindrical vector beam

## Abstract

Function expansion of fiber sensor is highly desired for ultrasensitive optical detection and analysis. Here, we present an approach of multifunctional fiber sensor based on Fano resonances and localized surface plasmon resonance (LSPR) excited via cylindrical vector beam with ability of refractive index (RI) sensing, nano-distance detection, and surface enhanced Raman spectroscopy (SERS). Silver (Ag)-nanocube modified microfiber is theoretically proved to enable to detect RI of the nearby solids and gases based on Fano resonances with a sensitivity of 128.63 nm/refractive index unit (RIU) and 148.21 nm/RIU for solids and gases, respectively. The scattering spectrum of the Ag nanocube has the red-shift response to the varies of the nano-distance between the nanocube and the nearby solid, providing a detection sensitivity up to 1.48 nm (wavelength)/nm (distance). Moreover, this configuration is theoretically verified to have ability to significantly enhance electric field intensity. Radially polarized beam is proved to enhance the electric field intensity as large as 5 times in the side-face configuration compared with linear polarization beam. This fiber-based sensing method is helpful in fields of remote detection, multiple species detection, and cylindrical vector beam-based detection.

## 1. Introduction

Function expansion of optical sensing is highly desired for different research field of biology, chemistry, physics, materials, etc [1,2,3,4]. Surface plasmon polaritons (SPPs) [5] have significantly promoted function expansion of optical sensing for its ability to trap light down to the subwavelength scale through noble metal or metallic nanostructures. Plasmonic Fano resonances and localized surface plasmon resonance (LSPR) are attractive phenomena brought by SPPs. Plasmonic Fano resonances are generated when metallic nanostructures are located at the vicinity of each other with small distance because hybridized bonding and antibonding plasmon modes arise [6,7,8], forming “bright” and “dark” modes [9,10,11], which coherently couple and interfere with each other and result in plasmonic Fano resonances [12,13,14,15,16]. LSPR [17] can be generated when metallic nanostructures have a size of sub-wavelength in three dimensions where SPPs are confined to three-dimensional bounded region.

Both Fano resonances and LSPR have shown great potential in optical sensing [18,19,20,21]. Fano resonances brought by symmetry-breaking structures like nanocubes on substrate [12], gold nano-disks integrated with indium tin oxide (ITO) [22], plasmonic nanoantenna on a graphene sheet [23], nanodisk surrounded by gold nanorods with different orientations [24], etc. often has high sensitivity to surrounding environment [25,26,27]. LSPR brought by different metal nanostructures i.e., nanoparticles [28], nanorods [29], nanostars [30] is also utilized in optical sensing for its ability of wavelength shift with the change of the surrounding [31] and enhancing scattering [32]. LSPR is significantly polarization-dependent [33,34,35], making it possible to improve the sensing performance through changing the illumination methods. When metal nanostructures are transferred onto the surface of fiber core and cladding, and the end-face and tip [36,37,38,39,40,41], a more multifunctional and reliable platform is promising to be built for the anti-interference, low noise, easy integration, and remote detection of optical fiber. Therefore, it is quite meaningful to investigate a new fiber sensing method based on plasmonic Fano resonances and LSPR under new-typed illumination.

Here, we present an approach of multifunctional fiber sensor based on Fano resonances and LSPR under cylindrical vector beam (CVB) illumination with ability of refractive index (RI) sensing, nano-distance detection, and SERS examination. A theoretical study is conducted on Silver (Ag)-nanocube modified microfiber for its capability of detecting RI of the nearby solids and gases based on Fano resonances with high sensitivity which is larger than that of multichannel fiber sensor based on Fano resonance and the recently-proposed metasurface RI sensor, and the ability of measuring the nano-distance between the nanocube and the nearby solid. Moreover, this configuration is theoretically verified to have ability of significantly enhancing electric field intensity in the case of radially polarized vector beam (RPB) excited side-face configuration with 60 nm-wide Ag nanocube, and in the case of linearly polarized beam (LPB) excited end-face configuration with the same nanocube, which can be further applied in SERS examination. The law of electric field enhancement excited by RPB, azimuthally polarized vector beam (APB), and LPB in side-face configuration and end-face configuration with different size and shape has been demonstrated. This fiber sensing method can be further exploited in remote detection potentially with improved multifunctionality and sensitivity.

## 2. Methods and Results

### 2.1. Refractive Index (RI) and Nano-Distance Sensor Based on Fano Resonances

**Figure 1:**
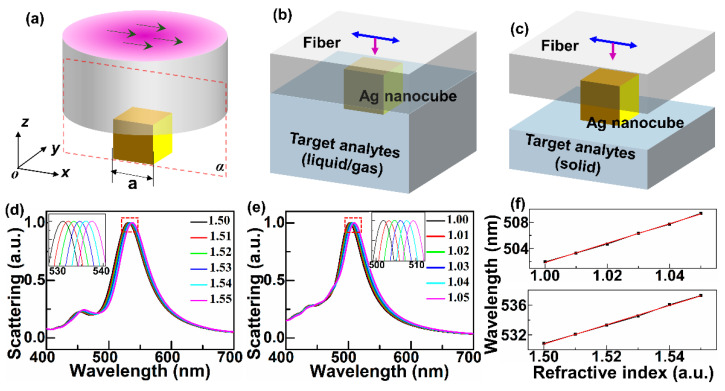
(**a**) Sketch map of microfiber integrated with an Ag nanocube on its end-face excited by LPB. (**b**) Sketch map of RI sensing of the gas surrounding by the Ag nanocube based fiber sensor. (**c**) Sketch map of RI sensing of the solid surrounding by the Ag nanocube based fiber sensor. (**d**) Normalized scattering spectra of the fiber sensor surrounded by solids with different RI of 1.50, 1.51, 1.52, 1.53, 1.54, 1.55, respectively. (**e**) Normalized scattering spectra of the fiber sensor surrounded by gases with different RI of 1.00, 1.01, 1.02, 1.03, 1.04, 1.05, respectively. (**f**) The peak position of the scattering spectra in (**d**) (bottom) and (**e**) (top) varies with the increase of RI (black) respectively and the corresponding fitted line (red).

Optical micro-fiber integrated with a silver nanocube on its end-face was investigated by a three-dimensional finite-difference time domain (3D FDTD-Lumerical, Ansys, Waterloo, ON N2J 4G8, Canada) method. The model of the fiber sensor is shown in Figure 1a, where the refractive index (RI) of the optical fiber is set as 1.48, and the wavelength of incident light ranges from 400 to 700 nm. The circular cross-section of fiber is regarded as an infinite plane in the simulation model considering that the fiber cross-section is much bigger than the nanocube length. Therefore, the simulation model of fiber in FDTD is abstracted to square shape as shown in Figure 1b,c. As exhibited in Figure 1b,c, the configuration of micro-fiber integrated with an Ag nanocube on its end-face is applied to detect the RI of the nearby gases and solids, respectively. The length of the nanocube is *a* = 60 nm. Ag is adopted as the nanocube material for its smaller ohmic loss in visible band [42], and described by Palik (0–2 μm) model in FDTD. A total-field scattered-field (TFSF) source is utilized as the illumination source with wavelength ranging from 400 to 700 nm. The PML boundary condition is chosen as the boundary condition of the simulation region.

Considering that solid analytes like glasses [43] have RI in range of 1.50–1.55, the scattering spectra of the Ag nanocube on the end-face of a fiber sensor surrounded by solids with different RI of 1.50, 1.51, 1.52, 1.53, 1.54, 1.55 are simulated out. The amplitude of the scattering cross-section changes with the change of the surrounding refractive index, leading to it unclear to see the wavelength shift. Normalization is carried out considering the wavelength shift of the normalized spectra being unvaried compared to the original spectra. Figure 1d shows the normalized scattering spectra of the Ag nanocube modified fiber sensor, the inset of which is the enlargement of the dotted box area. It can be seen from the inset that the spectrum is shifted with the increase of RI, and the corresponding curve of the peak position as the function of RI is exhibited in Figure 1f (bottom). The results in Figure 1f (bottom) reveals that the signal variation responding to the refractive index change of objects in the vicinity of the sensor surface has an excellent linear characteristic, which is important to be a valid sensing device. The calculated sensitivity that wavelength shift (nm) per refractive index unit (RIU) can be up to 128.63 nm/RIU. Moreover, the configuration is utilized to detect gases with different RI of 1.00, 1.01, 1.02, 1.03, 1.04, 1.05 for that a majority of gases like air and hydrogen [44] have RI close to 1. The normalized scattering spectra of the Ag nanocube surrounded by gases with different RI of 1.00, 1.01, 1.02, 1.03, 1.04, 1.05 are respectively calculated out, as shown in Figure 1e. Figure 1f (top) shows the peak position of the scattering spectra in Figure 1e varying with the refractive index (black) and the corresponding fitted line (red), which also demonstrates good linearity and sensitivity as high as 148.21 nm/RIU. The sensitivity is higher than that of multichannel fiber sensor based on Fano resonance (typically 22.9160 nm/RIU) [45], and the recently-proposed metasurface RI sensor (typically 110 nm/RIU) [46]. However, the sensitivity is lower than that of specialized approaches for RI sensing like gold nanowire modified fiber sensor (typically 12,314 nm/RIU [40]), photonic crystal fiber-based plasmonic sensor (typically 11,000 nm/RIU [47]), etc.

**Figure 2:**
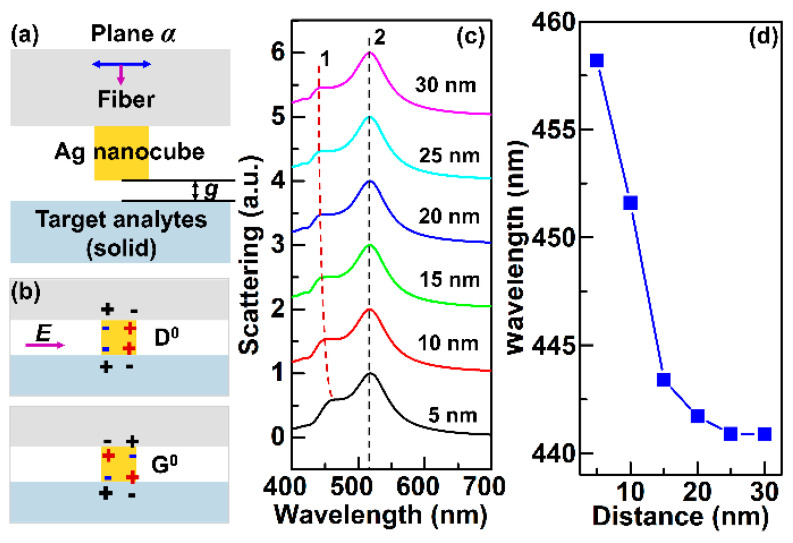
(**a**) Cross-section diagram of nanocube-based fiber sensor in plane *α* of Figure 1a for nano-distance detection. (**b**) Illustration of the nanocube-based D^0^ and Q^0^ mode. (**c**) Normalized scattering spectra of the fiber sensor surrounded by solid with different distances, keeping solid RI of 1.50. (**d**) The peak position labeled 1 of the scattering spectra in (**c**) varies with the increase of distance *g* between the nanocube and solid.

**Figure 3:**
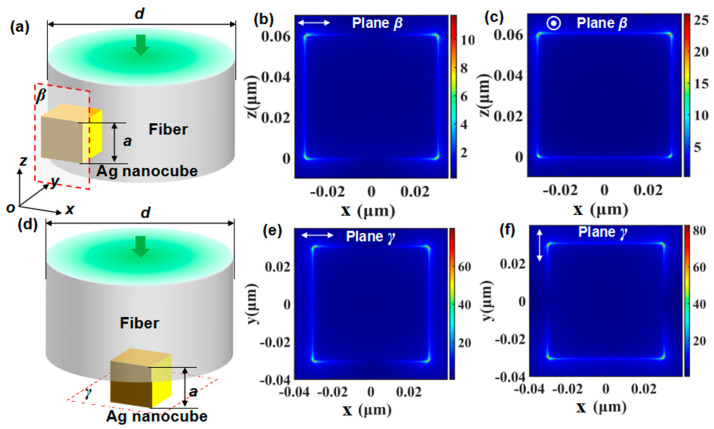
(**a**) Sketch map of microfiber integrated with a nanocube on its side-face excited by LPB. (**b**) Electric intensity distributions of plane *β* under excitation of LPB with polarization direction parallel to *x*-axis. (**c**) Electric intensity distributions of plane *β* under excitation of LPB with polarization direction parallel to *y*-axis. (**d**) Sketch map of microfiber integrated with a nanocube on its end-face excited by LPB. (**e**) Electric intensity distributions of plane *γ* under excitation of LPB with polarization direction parallel to *x*-axis. (**f**) Electric intensity distributions of plane *γ* under excitation of LPB with polarization direction parallel to *y*-axis.

Besides RI sensing, the fiber sensor can also be adopted to detect the distance in nanoscale. Figure 2a exhibits the cross-section in plane *α* of nanocube-based fiber sensor shown in Figure 1a for nano-distance detection. The scattering spectra of the nanocube are calculated out by 3D FDTD method, as illustrated in Figure 2b. It can be seen from Figure 2b that the wavelength location of peak 1 is red shifted with the decrease of distance *g* between the nanocube and solid, and that of peak 2 is constant with the change of *g*. It is mainly because primitive dipolar mode (D^0^) and primitive quadrupolar mode (Q^0^) shown in Figure 2b can interact with each other and generate hybridized bonding and antibonding mode^1^^2^, which can be influenced by the distance of the nanocube and the nearby solid. According to Ref. [12], the reason why peak 2 has no shift is that peak 2 is the antibonding mode originating from the Q^0^ mode. Peak 1 is the bonding mode originating from the D^0^ mode and can shift with the change of *g*. The intensity of peak 1 is much weaker than that of peak 2, which makes the peak shift not too visible in Figure 2c, but the peak shift is considerable and can be seen clearly in Figure 2d. Note that the wavelength shift of peak 1 responding to the change of distance g is nonlinear with g in range of 5–30 nm, as illustrated in Figure 3d, but in a small range from 5 to 15 nm, the responding curve is linear, and the sensitivity of which can up to 1.48 nm (Wavelength)/nm (Distance). It is of great potential in nano-gap measurement and forming a nano-ruler with advantages of high sensitivity, remote sensing, and reliability, which can be further applied in strain sensing, stress measurement, etc.

### 2.2. Electric Field Enhancement Based on LSPR

Apart from RI and nano-distance sensing based on Fano resonances, the nanocube integrated with fiber can be used to enhance electric field based on LSPR and further applied in SERS examination and sensing. Here, two kinds of commonly-used configurations, including side-face configuration (Figure 3a) and end-face configuration (Figure 3d) were theoretically investigated by 3D FDTD method, respectively. Figure 3a shows the simulation model of the side-face configuration excited by linear polarization beams (LPB) with the wavelength of 532 nm, where the diameter of the microfiber is *d* = 0.5 μm, and the side length of silver nanocube is *a* = 60 nm, and the plane *β* is coincident with one of the undersides of the nanocube that in the *x-z* plane. Figure 3d shows the simulation model of the end-face configuration excited by LPB, where the parameters of excitation wavelength, microfiber and nanocube are similar to that in Figure 3a, but the location of the nanocube is on the fiber end-face rather than side-face. The plane *γ* is coincident with one of the undersides of the nanocube that in *x-y* plane.

Figure 3b,c exhibit the electric-field intensity distribution of plane *β* excited by a pair of strictly degenerate vector modes with the polarization direction parallel to *x*- and *y*-axis respectively, which demonstrate that SPPs are excited on the edge of the Ag nanocube and LSPR is achieved at the vertex of the nanocubes, where the electric-field intensity enhancement induced by LPB with polarization direction parallel to *x*-axis is ~12, and that induced by LPB with polarization direction parallel to *y*-axis is ~25. It reveals that the electric field component perpendicular to plane *β* results in larger field enhancement than that of parallel direction in this side-face configuration. Figure 3e,f show the electric field distribution of plane *γ* excited by LPB with the polarization direction parallel to the *x*- and *y*-axis, respectively. It can be seen that significant electric-field intensity enhancement is achieved at the vertex of the nanocubes as high as ~70 and ~80 respectively by LPB with the *x*- and *y*-axis polarization direction. Noting that the polarization directions of these two degenerate modes of LPB are both parallel to the target plane labeled as *γ* in the end-face configuration, however, the enhancing magnification is much higher than that shown in Figure 3b,c. It is mainly because most of the energy on the cross-section of LPB is located at the center, which can easily reach the surface of the nanocube in the end-face configuration compared with the side-face configuration. Considering the field enhancement performance of Ag nanocube in the side-face and end-face configurations, it is promising to further apply these two configurations to the field of scattering-enhancement-typed fiber sensing like SERS to efficiently improve the sensitivity and obtain more information besides the RI information aforementioned by Fano resonances. In practical applications, silver nanocube can be fabricated via wet-chemical method [48]. Through combining with physical transfer method, the nanocube can be embedded on the end face or side face of fibers.

**Figure 4:**
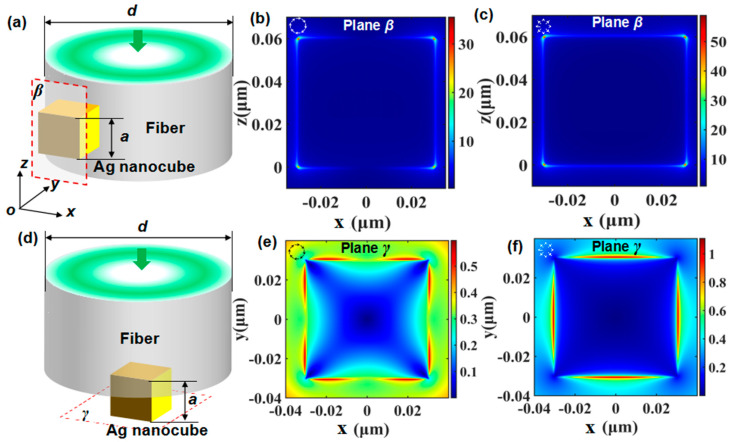
(**a**) Diagram of microfiber integrated with a nanocube on its side-face excited by CVB. (**b**) Electric intensity distributions of plane *β* under APB excitation. (**c**) Electric intensity distributions of plane *β* under RPB excitation. (**d**) Diagram of microfiber integrated with a nanocube on its end-face excited by CVB. (**e**) Electric intensity distributions of plane *γ* under APB excitation. (**f**) Electric intensity distributions of plane *γ* under RPB excitation.

The electric field enhancement can be notably influenced by illumination modes for the reason of the polarization-dependent excitation of localized surface plasmon modes [33,34,35]. Our previous work [36,37,38,49,50] demonstrates that cylindrical vector beams (CVB), including APB and RPB, are helpful to improve the field enhancement for their special polarization distribution on the cross-section of the light beam. Here, APB and RPB are adopted to excite the side-face configuration and end-face configuration, the simulation model of which is shown in Figure 4a,d, respectively. In the case of side-face configuration, Figure 4b,c show the electric field intensity distribution excited by APB and RPB, respectively, which is similar to that of LPB shown in Figure 3b,c, but the electric field intensity enhancement can reach ~35 and ~60 respectively, being much stronger than that excited by LPB. The improvement caused by APB and RPB compared with LPB results from the energy distribution characteristics and the polarization distribution characteristics on the cross-section of the light beam. The former reason refers to that most of the energy on the cross-section of APB and RPB are located near the fiber surface and that of LPB is located at the center, and thus leading to the evanescent field with different intensity reaching the nanocube. The latter reason is that APB and RPB have tangential and radial polarization distribution on the cross-section, which is also the origin of that RPB can obtain much higher electric field intensity enhancement compared with APB.

In the case of end-face configuration, Figure 4e,f show the electric field intensity distribution excited by APB and RPB respectively, revealing that LSPR is excited near the vertex of the nanocube but not at the vertex that like the case of LPB, and the electric-field intensity is lower than the input intensity of 1. In addition, in the case of RPB excitation, LSPR is excited at the center of the side surface of the nanocube, and not the vertex, but the electric-field intensity is a little bigger than the input intensity of 1, as exhibited in Figure 4f. Through comparing Figure 4a–f with Figure 3a–f, it can be seen that APB and RPB cannot always strengthen the electric-field intensity compared with LPB. The field enhancement performance depends on both the energy distribution and polarization distribution of the excitation light beam. The energy and polarization distribution characteristics can be consistent to improve field intensity or competitive to decrease field intensity with the change of the configuration of the incident light and nanostructures.

## 3. Discussion

### 3.1. Fano Resonances under Oblique Incidence

**Figure 5:**
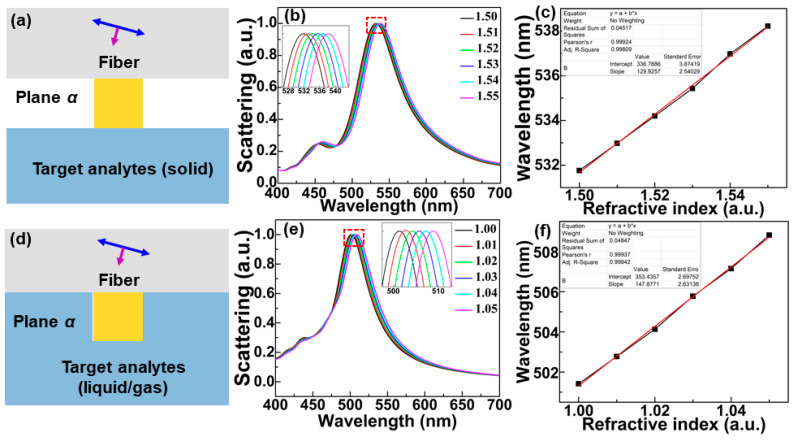
(**a**) Cross-section diagram of nanocube-based fiber sensor in plane α of Figure 1a for solid sensing under oblique incidence with a dip angle of 15°. (**b**) Normalized scattering spectra of the fiber sensor under oblique incidence surrounded by solids with different RI of 1.50, 1.51, 1.52, 1.53, 1.54, 1.55, respectively. (**c**) The peak position of the scattering spectrum in (**b**) varies with the increase of RI (black) and the corresponding fitted curve (red). (**d**) Cross-section diagram of nanocube-based fiber sensor in plane α of Figure 1a for gas sensing under oblique incidence with a dip angle of 15°. (**e**) Normalized scattering spectra of the fiber sensor under oblique incidence surrounded by gases with different RI of 1.00, 1.01, 1.02, 1.03, 1.04, 1.05, respectively. (**f**) The peak position of the scattering spectrum in (**e**) varies with the increase of RI (black) and the corresponding fitted line (red).

Fano resonances of a silver nanocube on the end-face of optical fiber under oblique incidence were discussed here. Figure 5a shows the cross-section diagram of the nanocube-based fiber sensor for solid sensing shown in Figure 1a under oblique incidence with dip angle of 15°. Figure 5b exhibits the normalized scattering spectra of the Ag nanocube surrounded by solids with different RI, and the inset is the enlargement of the dotted box area, which illustrates that the spectra are red-shifted with the increase of RI. The corresponding line of the peak position as the function of RI is shown in Figure 5c, revealing a good linear characteristic and a calculated sensitivity up to 129.90 nm/RIU. Moreover, in the case of gases detection, as shown in Figure 5d, the normalized scattering spectra of the surrounding gases with different RI have the same characteristics as the case of solid detection. It can be seen from Figure 5e,f that the spectra are red-shifted with the increase of RI, and the sensitivity is calculated to be 147.90 nm/RIU. Through comparing Figure 1d–f and Figure 5a–f, it can be seen that the sensitivity under normal incidence and oblique incidence is comparable, revealing that oblique incidence with dip angle of 15° cannot reduce the sensing performance.

### 3.2. Electric Field Enhancement Excited via CVB

**Figure 6:**
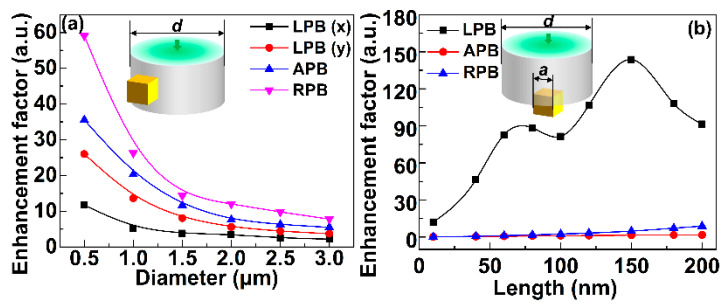
(**a**) Enhancement factor of electric field intensity induced by nanocube modified fiber on its side face excited via LPB, APB, and RPB respectively with the variation of the fiber diameter (*d* = 0.5, 1, 1.5, 2, 2.5, 3 μm). The size of the nanocube keeps constant of 60 nm. (**b**) Enhancement factor of electric field intensity induced by nanocube modified fiber on its end face excited via LPB, APB, and RPB respectively with the variation of the nanocube length (*a* = 10, 40, 60, 80, 100, 200 nm). The diameter *d* = 0.5 μm of the fiber remains unchanged.

The electric field enhancement can be notably influenced by illumination modes because the excitation of localized surface plasmon modes is polarization-dependent [33,34,35]. CVB including APB and RPB are efficient to improve the field enhancement based on their special polarization distribution [36,37,38,49,50]. Therefore, CVB is used to excite the side-face and end-face configuration. Electric field enhancement induced by silver nanocube modified optical fiber under LPB and CVB was discussed here. Figure 6 demonstrates the influence of the geometric dimensioning of side-face and end-face configuration on the electric field enhancement of these two configurations under CVB and LPB excitation. As illustrated in Figure 6a, the enhancement factor of electric field intensity induced by nanocube modified fiber on its side face excited via LPB, APB, and RPB respectively with the variation of the fiber diameter is calculated out, while the size of nanocube keeping constant of 60 nm. Note that no matter how the diameter of the fiber changes, the enhancement factor obtained via CVB keeps larger than that by LPB. But the enhancement factor under both LPB and CVB excitation dramatically decreases with the increase of the fiber diameter, which originates from the intensity changes of the evanescent field for its strong dependence on the diameter. Moreover, in the case of the end-face configuration, the enhancement factor of electric field intensity excited via LPB, APB, and RPB respectively with the variation of the nanocube length is shown in Figure 6b, where the diameter *d* = 0.5 μm of the fiber remains unchanged. It can be known that LPB generates a much bigger electric-field intensity enhancement compared with CVB, and the trend remains the same with the increase of the nanocube length. In view of CVB excitation, the enhancement factor is incremental with the increase of the length of the nanocube, but it is still smaller than that of LPB excitation. The increasing trend reveals that the energy distribution can affect the enhancing performance, which is consistent with the results in Figure 4. Figure 6a,b demonstrate that the electric field intensity enhancement can be improved via RPB as large as five times in the side-face configuration. And in end-face configuration, electric field intensity enhancement keeps more than 70-times higher under LPB excitation than that by APB and RPB excitation. CVB can efficiently strengthen the electric-field intensity compared with LPB only when the scale and shape of the metalized nanostructure and fiber meet the matching condition for the energy and polarization distribution characteristics can be consistent to improve field intensity or competitive to decrease field intensity with the change of the configuration of the incident light and nanostructures.

## 4. Conclusions

In summary, we present a multifunctional fiber sensor based on Fano resonances and LSPR with the potential of RI sensing, nano-distance detection, and SERS examination. Silver-nanocube modified microfiber is theoretically proved to detect RI of the nearby solids and gases based on Fano resonances. The simulation results show that the RI detection sensitivity can reach 128.63 and 148.21 nm/RIU respectively for solids and gases. The sensitivity is higher than that of multichannel fiber sensor based on Fano resonance (typically 22.9160 nm/RIU), and the recently-proposed metasurface RI sensor (typically 110 nm/RIU). The sensing performance keeps excellent under oblique incidence with a dip angle of 15°. In addition, the scattering spectrum of the Ag nanocube has considerable red-shift with varies of the nano-distance between the nanocube and the nearby solid, where the detection sensitivity can up to 1.48 nm (Wavelength)/nm (Distance) in a distance ranging from 5 to 15 nm. Moreover, this configuration is theoretically verified to have ability of significantly enhancing electric field intensity, which can be further applied in the SERS examination. The simulation results show that the electric field intensity enhancement can be improved via RPB as large as 5 times in the side-face configuration.

## Data Availability

Not applicable.
